# LB1528C. Ivermectin for Treatment of Mild-to-Moderate COVID-19 in the Outpatient Setting A Decentralized, Placebo-controlled, Randomized, Platform Clinical Trial

**DOI:** 10.1093/ofid/ofac492.1883

**Published:** 2022-12-15

**Authors:** Susanna Naggie

**Affiliations:** DCRI/ School of Medicine, Durham, North Carolina

## Abstract

**Background:**

The effectiveness of ivermectin to shorten symptom duration or prevent hospitalization among outpatients in the United States with mild-to-moderate symptomatic coronavirus disease 2019 (COVID-19) is unknown. We evaluated the efficacy of ivermectin 400 µg/kg daily for 3 days compared with placebo for the treatment of early mild-to-moderate COVID-19.

**Methods:**

ACTIV-6 is an ongoing, decentralized, double-blind, randomized, placebo-controlled platform trial to evaluate repurposed therapies in outpatients with mild-to-moderate COVID-19. Non-hospitalized adults age ≥ 30 years with confirmed COVID-19, experiencing ≥ 2 symptoms of acute infection for ≤ 7 days were randomized to receive ivermectin 400 µg/kg daily for 3 days or placebo. The main outcome measure was time to sustained recovery, defined as achieving at least 3 consecutive days without symptoms. Secondary outcomes included a composite of hospitalization or death by day 28.

**Results:**

Of the 3457 participants who consented to be evaluated for inclusion in the ivermectin arm, 1591 were eligible for this study arm, randomized to receive ivermectin 400 µg/kg (n=817) or placebo (n=774), and received study drug. Of those enrolled, 47% reported receiving at least 2 doses of SARS-CoV-2 vaccination. The posterior probability for any improvement in time to recovery was 0.91 (hazard ratio 1.07, 95% credible interval 0.96–1.17). The posterior probability of this benefit exceeding 24 hours was less than 0.01, as measured by the difference in mean time unwell. Hospitalizations or deaths were uncommon (ivermectin [n=10]; placebo [n=9]). Ivermectin at 400 µg/kg was safe and without serious adverse events as compared with placebo (ivermectin [n=10]; placebo [n=9]).

**Conclusion:**

Ivermectin dosed at 400 µg/kg daily for 3 days resulted in less than one day of shortening of symptoms and did not lower incidence of hospitalization or death among outpatients with COVID-19 in the United States during the delta and omicron variant time periods.

**Disclosures:**

**Susanna Naggie, MD/MHS**, AbbVie: Grant/Research Support|BMS/PRA: Event adjudication|Gilead Sciences: Grant/Research Support|Pardes Biosciences: Advisor/Consultant|Personal Health Insights, Inc: Data Safety Monitoring Board Member|Silverback Therapeutics: Advisor/Consultant|Vir Biotechnology: Advisor/Consultant|Vir Biotechnology: Stocks/Bonds.

